# Evaluation of Sentinel Lymph Node Dose Distribution in 3D Conformal Radiotherapy Techniques in 67 pN0 Breast Cancer Patients

**DOI:** 10.1155/2015/539842

**Published:** 2015-06-28

**Authors:** Gerlo Witucki, Nikolaus Degregorio, Andreas Rempen, Lukas Schwentner, Dirk Bottke, Wolfgang Janni, Florian Ebner

**Affiliations:** ^1^Diakonieklinikum Schwäbisch Hall, Abteilung für Strahlentherapie, Diakoniestr. 10, 74523 Schwäbisch Hall, Germany; ^2^Klinik für Frauenheilkunde und Geburtshilfe, Universitätsklinikum Ulm, Prittwitzerstr. 43, 89075 Ulm, Germany; ^3^Diakonieklinikum Schwäbisch Hall, Frauenklinik mit Brustzentrum und Genitalkrebszentrum, Diakoniestr. 10, 74523 Schwäbisch Hall, Germany; ^4^Klinik für Strahlentherapie und Radioonkologie, Universitätsklinikum Ulm, Prittwitzerstr. 43, 89075 Ulm, Germany

## Abstract

*Introduction*. The anatomic position of the sentinel lymph node is variable. The purpose of the following study was to assess the dose distribution delivered to the surgically marked sentinel lymph node site by 3D conformal radio therapy technique. *Material and Method*. We retrospectively analysed 70 radiotherapy (RT) treatment plans of consecutive primary breast cancer patients with a successful, disease-free, sentinel lymph node resection. *Results*. In our case series the SN clip volume received a mean dose of 40.7 Gy (min 28.8 Gy/max 47.6 Gy). *Conclusion*. By using surgical clip markers in combination with 3D CT images our data supports the pathway of tumouricidal doses in the SN bed. The target volume should be defined by surgical clip markers and 3D CT images to give accurate dose estimations.

## 1. Introduction

Over the last two decades the axillary dissection in early breast cancer has been replaced by the sentinel lymph node (SLN) biopsy [1, 2]. The physiology and techniques have been well described. The patients short and long term benefits are numerous [3].

The anatomic position of the sentinel lymph node is variable; therefore the surgeons mark the sentinel with blue dye or radioactive markers to locate it during surgery. To determine its location in relation to the standard radiation fields, surgeons can also place titanium clips. These clips can be identified on CT scans/X-ray. Several studies have described the correlation of axillary levels and sentinel nodes in relation to the spine or tangential radiation fields [4–9].

The benefit of tangential radiation on local disease control has been controversially discussed in the past [10]. With the technical advances made in radiotherapy planning and application it is now possible to distribute accurately an effective dose onto target tissue.

The purpose of the following study was to assess the dose distribution delivered to the surgically marked sentinel lymph node site by 3D conformal radiotherapy technique. We hypothesised that the standard breast tangent fields fail to deliver a dose of 40 Gy or more to the sentinel lymph node site.

## 2. Methods and Material

We retrospectively analysed 70 radiotherapy (RT) treatment plans of consecutive primary breast cancer patients with a disease-free, sentinel lymph node resection in the certified Breast Center Hohenlohe between January 2008 and August 2010.

One patient was excluded due to microinvasive cancer in the sentinel lymph node, one patient's primary cancer lump was too small to stain for hormone receptors after core needle biopsy (pT1a), and one patient was classified as pT4 with skin infiltration of the carcinoma. This left 67 data sets for analysis. SLN mapping was performed using a standard technique of perimamillary injection of* filtered* technetium-99 m-labelled human albumin colloid particles on the morning of the surgery. Mapping of the sentinel node was attempted with a gamma detecting camera and if unsuccessful repeated twice. Additionally after anaesthesia induction the patient was injected with blue dye perimamillary* (1 mL/quadrant = 4-5 mL)* by the surgeon.

The lymph node was identified with a gamma detecting probe and/or blue dyed lymph node(s). These lymph nodes were removed and two 5 mm titanium clips were placed to mark the location for this study. The standard pathology evaluation was done according to the S3-recommendations [4] in a certified pathological institute.

All patients underwent radiotherapy simulation with both upper extremities extended above their heads using an alpha cradle immobilization. Postoperative radiation was given by using a linear accelerator (Elekta Precise) from two (up to four) opposed tangential breast fields providing a cumulative radiation dose of 50 Gy photons as recommended by the ICRU (International Commission on Radiation Units) 50 reference point. Mixed energies of 6- and 10-MV photons were used in patients with large breasts. The therapy was administered over a 5-week period by using 2 Gy daily fractions and wedge compensators to achieve a uniform dose. The planned target volume encompassed the entire ipsilateral breast. The treatment field margins were determined by palpation of the breast parenchyma with the addition of a 2 cm margin in all directions and delineated with radiopaque markers. In general the superior margin was at, or near, the base of the clavicle, the medial margin near the midline, the lateral margin near the mid-axillary line, and the inferior margin 2 cm below the inframammary fold.

Additionally the surgical clips were identified on the images for the treatment plan. A virtual 10 mm circle was laid around the clips in the CT layer and a volume was calculated stretching the circle 5 mm in 3D. Descriptive statistics were calculated with SPSS for windows (version 19) and are given as means, standard deviation (SD), minimum (min), and maximum (max).

## 3. Results

A total of 67 consecutive patients were included in the study. The average age was 60 years with the youngest patient being 34 and the oldest 78 years old. On average 2-3 lymph nodes were removed (min-max: 1–7; SD 1.4).

42 patients were classified as pT1 and a further 25 as pT2. All patients had completed the surgical treatment with resection of the tumour with at least 0.5 mm resection margin.

The tumour characteristics and primary tumour site are displayed in Table 1. In our case series the SN clip volume received a mean dose of 40.7 Gy (min 28.8 Gy/max 47.6 Gy). In 42 patients (63%) the dose at the SN clips was at least 40 Gy, and 55 patients (82%) had a dose of 30 Gy or more. In eight patients the total dose of the SN was more than 50 Gy. In five of those the primary tumour was in the upper outer quadrant. A further two patients had the tumour located in the upper inner quadrant and one in the lower outer quadrant. This correlates with the distribution of the primary tumour sites. The radiotherapy data sets are illustrated in Table 2. The average follow-up time was 40 months (6–66 months). One patient died due to a nontumour related cause 40 months after diagnosis and one patient had a local and distant recurrence after 17 months. Primarily this patient (aged 79 years) had a hormone receptor positive tumour (ER12/PR8/Her2 negative) with lymphangiosis and minimum margins of 9 mm and 1 tumour-free sentinel node. The local recurrence occurred in the same breast but different quadrant. The sentinel bed received 46.4 Gy. The primary treatment was incomplete as the patient declined the recommended chemotherapy.

## 4. Discussion

Early breast cancer surgery has changed over the last two decades as the understanding of the disease has improved. The standard surgery has changed from mastectomy with axillary dissection to breast conserving and sentinel node biopsy improving the morbidity.

As the axillary dissection has failed to show any survival benefit [10–12], surgeons are starting to discuss the prognostic value of a lymphonodektomy (sentinel or total). In order to avoid morbidity the surgery could be replaced by neoadjuvant treatment, tumour genetics, or radiotherapy. Several trials [13–16] indicate that breast conserving radiotherapy has an effect on the axillary disease control. To the best of our knowledge this is the first time a virtual volume has been used around the clipped sentinel node location to give an accurate dose estimation.

The Canadian trial [14] reported a significant effect of breast radiation on axillary disease control. The study evaluated the radiation effect on HR+ tumours on women 50 years or older. The authors concluded that the radiation reduced the recurrence risk from 2.5 to 0.5%. The authors also note that the hormone receptors were not known in 13.3% of the patients and tamoxifen had to be stopped early in 243 out of 769 patients.

Similar results were published by Hughes et al. [15] looking retrospectively at patients aged 70 and over and positive estrogen receptors (ER). Radiotherapy (RT) did not have an overall effect on survival rates with tangential field radiation. A significant difference was found in the rates of local/axillary disease recurrence.

404 of the 636 patients did not receive a sentinel or axillary node dissection.

A prospective randomised controlled trial published by Veronesi et al. [16] looked at the radiotherapy of the axillary lymph nodes in women aged >45 and cN0. The authors found an overall lower recurrence rate than expected (total of 1%) and after a 5-year follow-up the RT group had 1 patient (0.5%) versus 3 patients (1.5%) with local recurrence. Due to the low numbers this effect was not statistically significant but according to the authors “Axillary radiotherapy seems to protect the patients from axillary recurrence almost completely.”

Finally an EBCTCG meta-analysis in 2005 analysed 17 trials with nearly 11000 patients and long term follow-up. This data showed a clear effect on the local and distant recurrence and long term survival [17] after radiotherapy. The study did differentiate between the type of surgery, histological grading, tumour size, ER status, antihormonal treatment, and lymph node status for the analysis, but no information regarding the radiotherapy technique was given. The authors point out that “screening, surgery, pathology, radiotherapy and systemic treatment have changed substantially since most of the women entered these trials.” Another trial showed no impact of chemotherapy sequencing on the local recurrence rate [18].

As Rabinovitch et al. [6] showed the variations of the anatomical location of the sentinel lymph node and the radiotherapy coverage of the sentinel lymph node in standard tangential radiation therapy fields do not encompass the lymph nodes at highest risk of containing tumour.

The authors also show that the sentinel lymph nodes would be included in the tangential fields design in 90% by removing the superior-posterior corner multileaf colluminator, similar to the results by McCormick et al. [19].

But these studies did not provide an explanation for the effect that tangential radiation has on axillary disease control. Still it is commonly assumed that in tangential field radiation the lower part of the axilla receives a tumouricidal radiation dose, but the tumouricidal dose has yet to be evaluated.

Krasin et al. [4] analysed 25 patient treatment plans and evaluated the level of breast treatment dose in the anatomy axillary levels. Only one patient received a dose of 50 Gy in the Level I axilla, and no patient had adequate coverage of the Level II or III axilla or the internal mammary lymph nodes. The study points out that the effective dose (50 Gy) might not be needed to show a clinical effect. The authors give three possibilities: (a) the tumouricidal dose is lower than that for the breast, (b) micrometastasis “on their way” are being eliminated, and (c) tangential radiation decreases the rate of subsequent seeding of the axilla.

Reed et al. [7] also published a dose distribution in the anatomical axilla volumes in 50 patients. Naturally in a subgroup of 18 patients undergoing axillary dissection they note a significant volume difference between the anatomical and the surgically marked axilla volumes (*p* < 0.001). In this subgroup 25% more patients had adequate dose coverage in the axilla.

Another study by Orecchia et al. [5] used a sentinel clip as definition of the caudal border in the anatomical defined axilla. Only one patient (out of 15) had an axilla dose of 40 Gy, but because the SN was used as caudal border the volumes were considerably smaller (Table 3). The location of the sentinel node might not be the most caudal lymph node of the axilla as shown by Rabinovitch et al. [6]. Nowadays it is possible to distribute an effective dose very accurately onto the breast tissue leaving other organs with minimal doses. With this technique the radiation dose on surrounding organs/anatomic structures (lung, heart and axilla) can be further reduced. As the tissue surrounding the sentinel node contains, if present, minimal residual tumour cells the target volume needs to be defined differently. A direct comparison of these studies is given in Table 3.

Contrary to the anatomical borders of the axilla we defined the target volume by clipping the sentinel bed intrasurgically and expanded a 10 mm circle around the clips. This circle was stretched 5 mm in the third dimension creating a target volume around the sentinel bed. This resulted in a mean dose of 40.7 Gy. Using only the clipped volume (i.e., sentinel location) our data shows that the location of the sentinel lymph node does receive a dose of 40 Gy or more in tangential breast radiation in most patients. This data supports the pathway of lower effective doses as mentioned by Krasin et al. [4]. Our dose results on target volume and lung show that the treatment was well delivered.

These results are in line with previous publications reporting an effect of breast tangential radiation on local tumour control [13–16]. As previously published [4] we found no correlation between the location of the primary tumour and dose distribution in the sentinel bed. Alongside current trials evaluating the general necessity of radiotherapy in breast cancer surgery (i.e., PROSPECTrial [20]), larger trials considering the tumour biology, accurate sentinel dose distribution, and long term follow-up are needed to define the effective dose.

From our point of view the target volume of the sentinel should not be defined by anatomical structures but by surgical clip markers and 3D CT images to give accurate dose estimations.

## Figures and Tables

**Table 1:** Histopathological tumour characteristics with T-classification according to the TNM system, ER = estrogen receptor; PR = progesteron receptor; SD = standard deviation.

	Number	
TNM		
Tis	0	
T1	42	
T2	25	
T3	0	
Total	**67**	
ER		
Positive	61	
Negative	6	
Total	**67**	
PR		
Positive	55	
Negative	12	
Total	**67**	
Her2 neu		
Positive (overexpression)	7	
Negative	60	
Total	**67**	

Margins	In mm	

Mean (SD)	4.6 (2.8)	
Min	0.5	
Max	10	

Location	Outside	Inside

Upper	37	20
Lower	5	5

**Table 2:** Radiotherapy information in Gy with mean dose, minimum (min) and maximum (max) doses. TV = target volume; SD = standard deviation; SLN = Sentinel lymph node.

	SLN min	SLN max	SLN mean	SLN SD
Mean dose	28.8	47.6	40.7	4.7
Min	1.56	3.1	2.4	0.2
Max	49.9	53	51.0	13.6
SD				12.4

	Lung min	Lung max	Lung mean	Lung SD

Mean dose	0.44	50.6	8.8	11.1
Min	0.11	47.441	6.471	8.3
Max	1.4	52.8	12.2	14.0

	TV min	TV max	TV mean	TV SD

Mean dose	3.3	53.6	47.7	5.4
Min	1.32	52.0	8.2	3.4
Max	5.2	53.9	49.6	11.0

**Table 3:** Literature review with details regarding planning mode, axillary findings, volume, and dose; n/g = not given; SN = sentinel node; ^*∗*^percentage of prescribed breast dose.

Author	*N*	Axillary region	Type of border (clip/anatomical)	TNM	Planning mode	Volume	Dose (mean value)
Orecchia et al. [5]	15	Level I	Anatomical (SN was clipped as caudal border)	N0 (sn)	CT-based	28.9 cm^3^	25 Gy

	50 total		Clip/anatomical		CT-based 3D	146.3 cm^3^ (anatomically)	n/g
Reed et al. [7]	*32 *	SN		N0 (sn)			
	*18 *	Level I & II		≤N1		104.8 cm^3^ (clip)	n/g

	106 total	SN	Clip		CT based		
Rabinovitch et al. [6]	*94 *			N0 (sn)		n/g	n/g
	*12 *			SN+		n/g	n/g

Krasin et al. [4]	25						
	*9 *	SN	Anatomical	n/g	CT based 2D	50 cm^3^ (anatomically)	32 Gy
	*16 *	Level I	Clip/anatomical	n/g			
						13 cm^3^ (clip)	12.9 Gy

	64				CT based		
Schlembach et al. [8]	*26 *	SN	Clip	n/g		n/g	98%^*∗*^
	*39 *	Level I & II	Clip	n/g		n/g	98%^*∗*^

SN+ = positive sentinel node; *N* = number of patients.
